# Clinical and Genetic Characterization of a Cohort of Small-for-Gestational-Age Patients: Cost-Effectiveness of Whole-Exome Sequencing and Effectiveness of Treatment with GH

**DOI:** 10.3390/jcm13144006

**Published:** 2024-07-09

**Authors:** Ramón Arroyo-Ruiz, Cristina Urbano-Ruiz, María Belén García-Berrocal, Elena Marcos-Vadillo, María Isidoro-García, M. Montserrat Martín-Alonso, Ana Fe Bajo-Delgado, Pablo Prieto-Matos, Juan Pedro López-Siguero

**Affiliations:** 1 Pediatrics Department, Reference Unit for Rare Diseases DiERCyL, University Hospital of Salamanca, 37007 Salamanca, Spain; rarroyoruiz@saludcastillayleon.es (R.A.-R.); curbano@saludcastillayleon.es (C.U.-R.); 2 Biomedical Research Institute of Salamanca IBSAL, 37007 Salamanca, Spain; mbgarcia@saludcastillayleon.es (M.B.G.-B.); emarcosv@saludcastillayleon.es (E.M.-V.); misidoro@saludcastillayleon.es (M.I.-G.); mmmartin@saludcastillayleon.es (M.M.M.-A.); 3 Clinical Biochemistry Department, Reference Unit for Rare Diseases DiERCyL, University Hospital of Salamanca, 37007 Salamanca, Spain; 4 Department of Medicine, University of Salamanca, 37008 Salamanca, Spain; 5 Pediatrics Department, Endocrinolgy Unit, University Hospital of Salamanca, 37007 Salamanca, Spain; afbajo@saludcastillayleon.es; 6 Department of Biomedical and Diagnostic Sciences, University of Salamanca, 37008 Salamanca, Spain; 7 Department of Pediatric Endocrinology, University Hospital of Málaga, 29010 Málaga, Spain; lopez.siguero@gmail.com; 8 Biomedical Research Institute of Málaga IBIMA, 29590 Málaga, Spain

**Keywords:** short stature, small for gestational age, exome sequencing, growth hormone, puberty, adult height

## Abstract

**Background/Objectives:** Develop a clinical and genetic characterization, in a group of small-for-gestational-age (SGA) patients who did not experience catch-up growth **Methods:** In an ambispective cohort study with (SGA) patients. These patients received one treatment with growth hormone (GH) over 14 years. This study analyzes their response to treatment and conducts a genetic analysis in order to identify cases with specific phenotypic and auxological characteristics, defined as presenting two or more dysmorphic traits and/or a stature below −3 SDS (standard deviation score). Whole-exome sequencing (WES) was performed on selected patients. **Results**: Forty-four SGA patients were examined, with an average age of 6.4 (2.49) years and an initial size of −3.3 SDS. The pubertal growth was 24.1 (5.2) cm in boys and 14.7 (4.3) cm in girls. WES in 11 SGA patients revealed conclusive genetic variants in eight, including two pathogenic ACAN variants, one 15q26.2-q26.3 deletion, and four variants of uncertain significance in other genes. **Conclusions**: Treatment with GH in SGA patients was shown to be effective, with a similar response in the group with positive genetic results and in the group who did not undergo a genetic study. Genetic testing based on auxological and clinical criteria proved highly cost-effective.

## 1. Introduction and Objectives

The term “small for gestational age” (SGA) was coined in 2001 by the International SGA Advisory Board Consensus Development Conference and the meeting that was celebrated in 2007 refers to newborns with a length and/or weight at birth that is 2 or more standard deviation scores (SDS) below the average for their gestational age (GA) [1,2], based on their population of reference, considering gestational age and sex. This definition, therefore, makes it possible to classify children with low weight, short stature, and both a low weight and short stature as SGA. In our context, tables and figures from the 2010 Spanish cross-sectional growth study are used to estimate the standard deviation score (SDS) [3,4].

The persistence of short stature in these patients is one of the most common complications in SGA patients. In most cases, there is catch-up growth by 2 years of age [2]. However, some patients do not present it and have short stature in their adult life.

Treatment with recombinant human growth hormone (GH) was approved for these patients in 2003 by the European Medicines Agency (EMA) [1], and it was subsequently implemented in Spain, where it accounts for 21% of all authorized treatments [5]. Its effectiveness has already been proven [6], but the causes for the significant heterogeneity in the responses has not been fully documented nor explained.

From an etiological standpoint, the causes of SGA are multiple. However, advances in genetics and the evolution of current techniques have increased the number of diagnoses of monogenic syndromes in SGA patients. The development of array comparative genomic hybridization (aCGH) and massive sequencing studies have made it possible to detect variants and other alterations with greater sensitivity [7]. However, and in spite of these advances, the monogenic etiology of SGA patients is still unclear in many cases [8,9]. Consequently, these tests become particularly relevant to detect unknown candidate genes and to expand on the information that is already available.

Unknown genetic alterations in SGA patients may be one of the causes that lead to variation in the response to treatment with GH. Due to their relatively high frequency and their potential repercussions, it is essential to conduct studies on the genetic causes that help us better classify those SGA cases that are still considered idiopathic in order to understand the way in which these genetic alterations may affect the response to treatment with GH.

The main goal of our study is to develop a clinical and genetic characterization, through new-generation sequencing techniques (whole-exome sequencing), in a group of SGA patients who did not experience catch-up growth and required treatment with GH. The secondary objectives are analyzing the response to the treatment among SGA patients and its safety, as well as the variables that can affect the patients’ response.

## 2. Patients and Methods

### 2.1. Study Design and Patient Selection

An ambispective study was designed in which a genetic analysis with whole-exome sequencing was performed on a selection of SGA patients. Retrospectively, a series of auxological variables were collected from the patients included in this study, and prospectively, genetic studies were conducted on selected patients based on the obtained variables.

The study was conducted in a regional reference unit data from all the children with SGA diagnosis who did not present catch-up growth and started treatment with GH, and who were monitored between 2008 and 2022.

The inclusion criteria were as follows: patients diagnosed as SGA at birth (height or weight below −2 SDS); not having experienced catch-up growth by age 4 years; and the start of treatment with GH that meets the criteria established by the European Medicines Agency and having a complete description of their phenotype.

After establishing the inclusion criteria, exclusion criteria were described as not meeting some of the inclusion criteria or presenting incomplete data.

**Figure 1:**
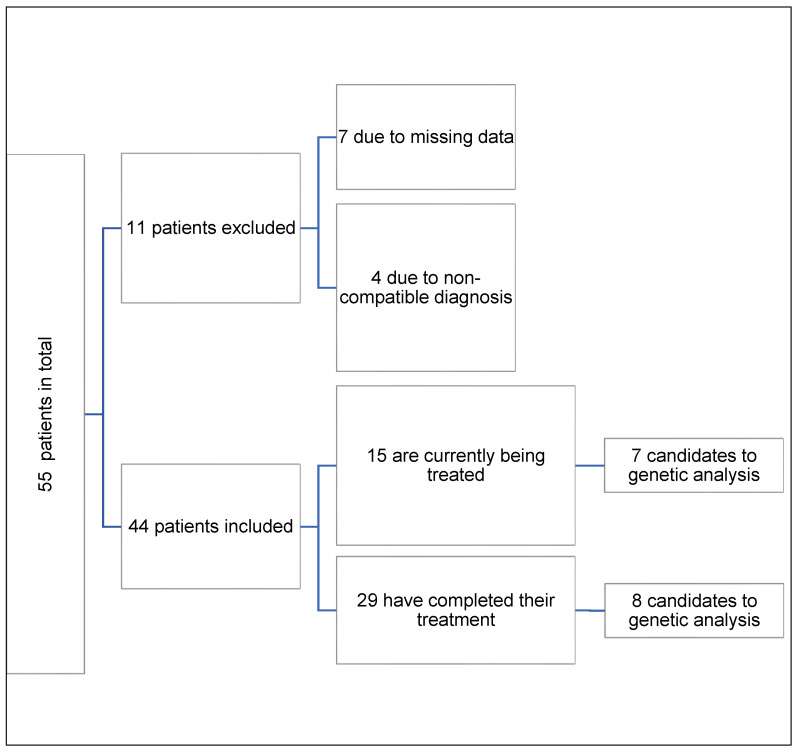
Patients in the study.

After searching the database containing a total of 494 children with short stature using the established criteria and analyzing the clinical history of the patients according to the inclusion and exclusion criteria, 44 SGA patients were identified that could be included in this study (Figure 1).

### 2.2. Methods

The initial clinical and auxological variables selected for the study were the date of birth, sex, gestational age, weight (SDS), and height (SDS) of the newborn, height of the parents, and target height (SDS).

In addition, the following variables were registered at the start of the treatment: age, weight (SDS), height (SDS), and complete description of the patient’s phenotype. Finally, the monitoring variables included the chronological age in each visit, height (SDS), weight (SDS), pubertal status, GH dose (mg/kg/day), IGF-1 (ng/mL), insulinemia (µIU/mL), bone age, and growth rate (cm/year).

The final variables were adult height, defined as the height reached when the growth rate was lower than 2 cm/year, total height gain (which was calculated as adult height − height at the start of GH treatment), and total pubertal gain (calculated as adult height − height at onset of puberty).

### 2.3. Genetic Studies

Once the SGA population had been defined, patients with particular clinical characteristics were selected, their DNA was collected, and a complete genetic study was conducted for those patients.

The genetic study was conducted on SGA patients who were selected due to phenotypical traits that were not part of a specific syndrome and patients whose initial height was affected more significantly.

Out of all the SGA patients included in this study, 15 were selected for genetic analysis. Out of these 15 patients, in 3 cases, it was impossible to get in contact with them to obtain samples.

The genetic study was conducted via whole-exome sequencing in all the patients who met the required criteria. Prior to the sequencing, a karyotype was obtained for girls, and their SHOX/PAR1 region was screened using the MLPA technique.

The DNA extraction was conducted from peripheral blood samples with the MagNaPure Compact system (Roche Diagnostics (Rotkreuz, Switzerland)). The DNA libraries were created with TruSeq Exome Library Prep technologies (Illumina (San Diego, CA, USA), and the whole exome was captured with xGem Exome Research Panel (Integrated DNA Technologies, IDT (Coralville, IA USA)). The massive sequencing technology NextSeq 500 (Illumina) was used to sequence the libraries. The obtained data were analyzed through the platforms DNAnexus, Saphetor Varsome and Datagnomics and aligned with the human genome reference GRCh37/hg19. Finally, the variants obtained were classified according to the American College of Medical Genetics and Genomics Standards and Guideline as “pathogenic”, “likely pathogenic”, “uncertain significance”, “likely benign”, and “benign”.

### 2.4. Statistical Analysis

The statistical study was conducted with SPSS 17.0. Prior to the analysis, all the data were subject to the Kolmogorov–Smirnov test to determine whether the variables had a normal distribution. In order to compare the variables with normal distribution, Student’s *t*-test was used for paired or independent samples. In the case of variables without normal distribution, the U Mann–Whitney test was used for independent samples and Wilcoxon’s test was used for paired samples. Whenever it was necessary to compare several groups, ANOVA with Bonferroni correction was used for variables with a normal distribution and Kruskal–Wallis test was used for the rest.

Statistical significance was established for *p* < 0.05.

### 2.5. Ethical Aspects

Prior to conducting the study, approval from the Research Ethics Committee of the Hospital was requested and obtained with the CEIm reference code: PI 2021 04 669.

All data were considered confidential and no personal information appeared on the database. The patients were indexed according to their clinical history number, and the database was only visible for the researchers involved.

This study meets the principles established by the WMA Declaration of Helsinki (52nd WMA General Assembly, Edinburgh, Scotland, 2000) and the good clinical practice guidelines, and it complies with the Spanish laws regulating biomedical research on humans (Law 14/2007 on Biomedical Research)

## 3. Results

### 3.1. Descriptive Analysis

A complete analysis was conducted for all SGA patients who were monitored by our department, which accounted for a cohort of 44 SGA children (19 boys and 23 girls) who met the established criteria, 29 of whom reached adult height.

The initial and evolutionary descriptive variables are presented in Table 1 and Table 2.

A comparison between sexes shows that boys started treatment with an average height (SDS) of −3.1 (0.48), whereas in girls, the average height was −3.5 (0.7), without statistically significant differences. No significant differences were observed between the sexes regarding height at birth, weight at birth, the age of treatment initiation or height at the start of treatment.

The greatest height gain was observed after 1 year of treatment, and it was higher in boys than in girls, although the differences were not statistically significant.

After 3 years of treatment, the gain was still slightly higher in boys, which leads to a greater height in the third year of treatment in this group, which was −2.02 (1.08), without statistically significant differences.

SGA patients started puberty at an average age that was adequate both for their age and their sex: 10.48 years (1.16) for boys and 9.94 years (0.8) for girls, without any registered cases of precocious puberty. If we compare these data with the study carried out by Carrascosa [10], 72% of our patients could be considered early or very-early maturing, despite reaching their pubertal growth spurt at an adequate age, which can generally indicate a precocious pubertal onset.

The height at the start of puberty was much higher than at the start of treatment, with an average of −1.3 SDS (0.97). No statistically significant differences were observed regarding sex.

The height gain during puberty was 24.1 (5.2) cm in boys and 14.7 (4.3) cm in girls. This pubertal growth was lower than that of the reference population in the current existing longitudinal population studies [4]

Data for pubertal variables are shown in Table 3 and Table 4.

Adult height (SDS) in boys was −1.2 (1.03), whereas in girls it was −1.83 (1.16), and the differences were not statistically significant.

In our study, 76.3% of the patients reached normal height, and only 23.7% were below −2 SDS. When comparing patients by sex, we observed a significant difference between boys and girls, because height normalization was reached in 78.6% of the boys vs. 61.1% of the girls (*p* < 0.05).

**Figure 2:**
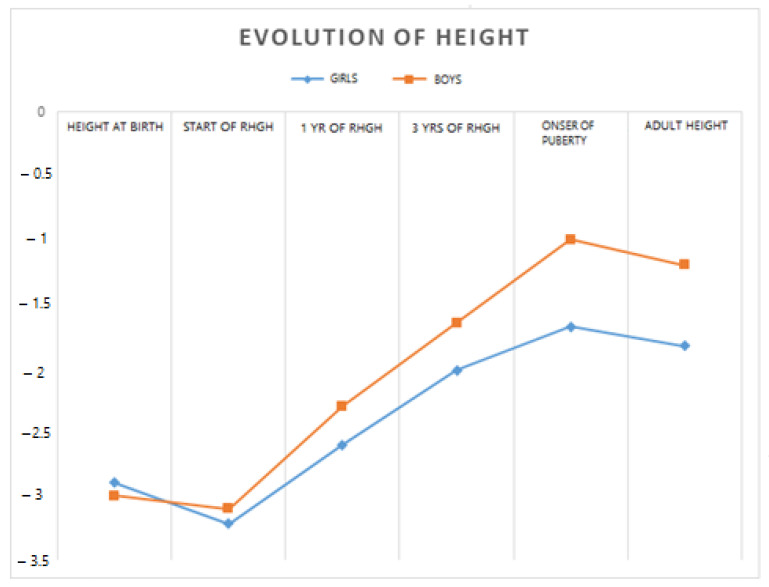
Evolution of height compared by sex.

Figure 2 shows the evolution in the height of patients by sex.

The total height gain in our patients was an average of 1.69 SDS (0.88), and these values were very similar for boys and girls.

### 3.2. Inferential Analysis

Table 5 shows the correlation of the analyzed variables with regard to the height and height gain in SGA patients.

### 3.3. Adverse Events

The adverse effects that are potentially related to the treatment were very few and mild, and the treatment was very well tolerated by the patients.

In one of our patients, an alteration of the glycemic metabolism was observed, which improved spontaneously without requiring an interruption of the treatment. In the rest of the cases, no adverse events were registered.

### 3.4. Genetic Analysis

From the pool of 494 patients with short stature, after selecting 44 eligible candidates for the genetic study, 11 exome analyses were conducted. This decision was influenced by one patient being diagnosed via karyotype prior to analysis. The patient exhibited a complete deletion of the X chromosome and was diagnosed with Turner syndrome, despite lacking typical clinical symptoms.

Out of the 11 patients who underwent the study, 8 of them presented a genetic variant which was thought to be associated with their symptoms, while in the remaining 3 cases, no variant was identified that could justify the patients’ symptoms.

Table 6 summarizes the initial auxological data of the patients in which genetic variants were observed that accounted for their symptoms. Most of the patients who have reached their adult height have also reached their target height. In spite of not having normalized their height, it was very close to their target height.

Out of the eight variants that were identified (Table 7), three were pathogenic, one was likely pathogenic, and four had uncertain significance.

Patients SGA-1 and SGA-2 are siblings and present an *ACAN* variant. They started treatment with growth hormone at 9.2 years and 4.3 years, respectively, with a good response to the treatment and a height gain of +1.5 SDS, compared to the beginning of the treatment. After the genetic analysis of the patients, a segregation study revealed that the variant was of paternal origin. The father of the patients had received treatment with GH during his childhood. It started at age 8.8 and he presented a very marked short stature, which was −4.2 SDS. Currently, the father shows a disproportionately short stature of 159.8 cm (−2.7 SDS) with an arm span of 174.5 cm, together with symptoms of osteoarthritis. Despite this, the response to GH for both the father and patient SGA-1 was good.

The clinical alterations observed in patient SGA-4 could be explained by a variant identified in the *IHH* gene. This patient presents a particular phenotype, with genu valgum, cubitus valgus, the bilateral clinodactyly of the fifth fingers, camptodactyly and slight broad pectus excavatum. After analyzing the patient, the study was made to his sister and his father, who also presented short stature and the same variant.

Patient SGA-5 (variant in the *FGFR3* gene) presents short stature, which was not very marked at birth (−2.8 SDS), and the only phenotypical trait is a triangular face. If this had been found to segregate across multiple generations, the variant would have been classified as likely pathogenic. However, since it was also found in her father (height: −1.6 SDS), it was classified as having uncertain significance.

Patient SGA-6 showed a variant of uncertain significance in gene *GHR*, which was neither clinically nor analytically compatible with his symptoms. However, given the description of cases of short stature and isolated variants of the *GHR* gene described in the literature, it was considered the cause of the disease.

In patient SGA-7, two variants of uncertain significance were found: one mutation in the *GHR* gene, which could be somehow involved in the patient’s height, and another one in the *ACAN* gene, which could more accurately account for the symptoms of short stature and advanced bone maturation without osteoarthritis.

Finally, patient SGA-8 presented a del15q26.2-q26.3 variant, and the examination revealed general hypotonia, hypertelorism, depressed nasal root, low-set ears, epicanthus without ptosis, micrognathia, and syndactyly between the second and third toes in both feet. These signs, together with the patient’s height, led to the conclusion that the uncertain significance variant of the patient matches the diagnosis.

## 4. Discussion

### 4.1. Genetics

This study shows the high cost-effectiveness of genetic analyses, as long as they are conducted after careful clinical and auxological selection of the patients. It also reveals how genes associated with growth cartilage are more often found in patients that are small for their gestational age.

We know that the genetic causes of SGA represent 19% of the patients born with intrauterine growth restrictions. However, the incidence single of nucleotide variants or of a number of copy variants is not properly established [11].

In our research, 72.7% of the results matched the clinical symptoms. However, when focusing on pathogenic variants, only 27.2% of the cases were identified. These results are due to a very detailed selection of patients, which is at the same time, an advantage and a limitation when conducting genetic analyses in small-for-gestational-age patients.

While our study was based on whole-exome sequencing, techniques for genetic diagnosis are very varied, and the analysis of copy number variations is becoming an increasingly used genetic tool nowadays. Given that the genetic etiology of short stature is multifaceted, involving copy number variations (CNVs), single-nucleotide variations (SNVs), as well as methylation, it is imperative to implement other techniques capable of analyzing these diverse types of variants. Integrating such methods into genetic analyses can provide a more comprehensive understanding of the genetic underpinnings of short stature and enable more accurate diagnosis and personalized treatment approaches. For example, Hui Zhu et al. conducted a study with array CGH on 107 FGR cases in which they found 18.8% of pathogenic variants [12]. For their part, Canton et al. [13] analyzed 51 SGA patients with array CGH, with an effectiveness of 16%.

Conclusive results are more frequent when diagnosis is reached via exome sequencing and after a careful selection of the sample. The study by Toni et al. [14] analyzed a panel of 399 growth-related genes by whole-exome sequencing in 182 patients, without a selection based on clinical criteria, and they found variants that matched the symptoms in 40 of the cases. These results were similar to those by Park SJ. et al. [15], with 47.6% conclusive results and a lower proportion of pathogenic or likely pathogenic variants. In that study, the genes that conditioned the symptoms, unlike the rest, were not associated with growth cartilage, perhaps because the study was conducted without neonatal selection.

Finally, and with regard to cost-effectiveness, the research by E. Inzaghi et al. [16] on a cohort of Italian patients has similar results to ours. Their group also conducted a genetic analysis after a selection with clinical and auxological criteria, and they found single-nucleotide variations in 14/28 (58%) of the patients.

**Figure 3:**
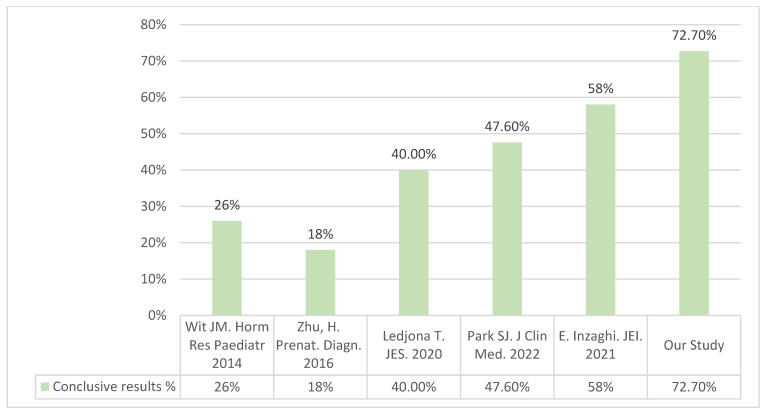
Comparison of effectiveness of molecular diagnostics in international studies [8,12,14,15,16].

Figure 3 shows a comparative summary of these studies, which provides a graphic representation of the improved effectiveness after an adequate genetic study with patients selected based on clinical criteria.

From the perspective of genetic characterization, most of the variants in our patients were associated with the growth cartilage (*IHH*, *ACAN*, *FGR3*…). The *ACAN* gene was the most common finding in our study, and it was present in 37.5% of all patients with a positive genetic analysis; however, it is worth noting that two of the patients are siblings. These results are similar to those in the study by van der Steen et al. [17], in which exome sequencing was performed in 29 patients selected from a group of 290 children who had been SGA at birth and had advanced bone age. In their study, 13.8% of those patients presented variants in the *ACAN* gene.

While our patients only presented advanced bone age with symptoms of osteoarthritis, in one case, a study found classic phenotypical characteristics such as midface hypoplasia, joint disorders or broad great toes and advanced bone age in a parent with severe short stature that was <−3.5 SDS. When patients presented these specific clinical features, the effectiveness of the genetic study was virtually 100%. One of the variants found in their study was a missense in exon 10, very similar to what was observed in our patient SGA-7.

With regard to the case of our patient SGA-6, we found a variant in exon 6 of the *GHR* gene, which is different from the classic deletion that leads to a poor response in GH treatment, and which is generally found in exon 3 [18]. The latest studies, however, have already identified multiple variants in *GHR*, and more specifically in SGA patients, which cause inadequate sensitivity to GH [19,20].

Another example are variants in *IGF1R*, which have also been reported as the etiology for SGA cases [20,21], as in patient SGA-8 in our study. The copy number variation presented by the patient is one of the most common in children who are SGA. According to the current literature, it is the second most common CNV in SGA patients, and it is found in 9.2% of patients who are genetically SGA due to CNV [13,22,23,24,25].

We cannot currently determine the variants that will affect the response to treatment, or those that cause intrauterine growth restrictions. However, the publication of an increasing number of studies on this matter is creating a repository of data and variants that make it possible to conduct a more comprehensive analysis of patients with these mutations, and to provide better clinical assessment for their condition.

This reinforces the fact that the genetic assessment of SGA patients is complex, and that a periodic reassessment of their original diagnosis is extremely important due to the broad heterogeneity of cases and the need for precision medicine.

### 4.2. Response to GH

Our research shows once again the effectiveness and safety of GH treatment in SGA patients. However, we were not able to determine which variants affect the response to treatment. We observed a positive correlation between increased height gain at the third year and final adult height (r = 0.4) (*p* = 0.048), and a negative correlation between the age at start of treatment and the total height gain (r = −0.6) (*p* = 0.3). Since not a large number of statistically significant results has been found, the multivariant analysis that was initially planned to create a predictive model will be postponed for a future study when more data on patients with adult height are available.

The adult height reached by the patients in our study is slightly higher than what was observed in other national studies [6,26], although it was lower than what was observed in the study with the largest sample [5]. We observed a shorter adult height in girls than in boys, but without statistically significant differences between them. Although the adult height was lower, the total height gain during treatment was very similar for both sexes.

The common aspect in these heterogeneous findings is the fact that our SGA patients suffered from height loss during puberty, caused by a reduced pubertal growth spurt compared with the general population defined in Spanish longitudinal studies [4,27] or even with patients affected by other conditions, such as idiopathic short stature or short stature caused by GH deficiency [28].

In current research, and on the contrary to what could be expected, female patients present a pubertal gain that is significantly lower than that in the reference population, unlike in the study previously published by our team [29] or other studies such as the one by Campos Martorell et al. [26]. However, this marked height loss during puberty is once again found in both men and women and, even though it is not as marked as in the previously analyzed series, men present a greater adult height loss compared to their height at the onset of puberty than women.

Puberty onset was observed earlier in our current series than in the reference population, particularly for girls. This had not been the case in our previous study [29], and it may account for the difference in pubertal height gain observed in girls, although this finding has also been reported by other research groups [30]. It is also important to point out that our inferential analysis found a negative correlation between the age of puberty onset and the height gain during puberty, even if it was not statistically significant (r = −0.35) (*p* = 0.051).

This may also be due to the fact that the average age at the start of treatment in our patients was higher, since some recent studies have shown that starting at ages 2–4 years leads to an increased height gain [31]. However, given that patients present higher levels of IGF1, this may also lead to precocious puberty.

The fact that we have found once again this loss in pubertal gain reinforces the hypothesis that the treatment with GnRH agonists can represent a significant improvement in the adult height of SGA patients, even in cases in which the height prognosis is below −2.5 SDS at the onset of puberty. The combined treatment with GnRH and GH has been shown to be beneficial in improving the height gain, particularly in patients who started treatment with GH late for different reasons [32], and with better results than increasing the isolated dose of GH, which has not shown good results [33,34]. This approach is not only safe and effective, but there have been no metabolic complications that justify not using it in cases in which it may benefit the patient. Different longitudinal studies [35,36] have shown that a combination of GH treatment for two years and the continuous use of GnRH agonists does not seem to have long-term negative effects on body composition, insulin resistance, plasma lipid levels, or even arterial pressure. This beneficial impact lasts up to five years after suspending GH administration. Even in cases in which girls present catch-up growth, there may be a significant metabolic effect during puberty [37], treatment could not also help with height, but also prevent undesired metabolic effects that are observed in these patients.

Finally, when comparing the response to treatment in the group who underwent a genetic analysis and in the group who did not, we observed that the response to treatment was similar with regard to height gain, in line with recent articles, such as the Belgian study [38], which observed that adult height was lower in syndromic than non-syndromic SGA patients: −2.59 SDS (−4.99/−1.57) vs. −2.32 SDS (−3.3/−1.2), but with very similar height gain in both groups: +0.76 (−0.70/1.48) for syndromic patients, and +0.86 (−0.12/1.86) for non-syndromic patients, without statistically significant differences. The Italian study [16] also observed good response among patients with conclusive genetic studies, but only provided data up until the first year of treatment.

If we analyze the specific case of our patients with variants in the *ACAN* gene that affect their symptoms and compared them with the patients in the study by van der Steen [17], we can observe that, in both cases, the response to treatment with GH was satisfactory. One fundamental difference that led to better results in their work is that their patients were treated with GnRH analogs for at least two years, and in the case of boys, they started a subsequent treatment with aromatase inhibitors, which led to an increased height gain of 7–8 cm in patients who reached adult height. This improved stature strengthens our previous hypothesis that treatment during puberty should be reinforced, even with aromatase inhibitors in boys, since there are already published studies that start this treatment to improve stature.

These findings lead to the supposition that GH treatment may be effective even in patients with the syndromic trait, and that genetic characterization is particularly relevant, since it makes it possible to guide the treatment better and to intensify it during puberty if necessary, with a combined treatment with GhRH analogs. Unfortunately, the data from our study do not make it possible to determine which variants could affect the response to treatment or cause intrauterine growth restrictions. However, the publication of an increasing number of studies on this topic is leading to the creation of a repository of data and variants that will allow a more comprehensive analysis of patients with these mutations and offer them better clinical counsel on their condition.

## 5. Study Limitations

The main limitation in our research, as well as in similar studies analyzing the long-term response to GH, was the reduced number of patients who reached their final height, which limits the capacity to reach conclusions regarding height gain, and to establish a comparison between groups. This has also meant that the statistical power of our study may be limited.

The availability to conduct whole-exome sequencing is limited, which also affects our capacity to carry out whole-exome sequencing in all the patients in our sample and made it necessary to carry out a preliminary study to select specific patients. This might create a selection bias that could affect our conclusions.

Another limitation may be that the indication for growth hormone therapy in patients with dysmorphic traits might be subject to debate. However, since they did not present clear facial gestalt, and their height was severely affected, treatment was considered beneficial for the patients when it was started.

## 6. Conclusions

The clinical characterization of patients who are SGA and the analysis of their genetic profile is essential. Even though these groups of patients present common symptoms, the identification of specific genetic variants may be fundamental, particularly when we observe a positive response to growth hormones in individuals with a specific phenotype.

Examining and knowing the genetic variants that condition that phenotype will allow us to predict whether the response to hormone growth will be positive or negative, and this will largely improve the quality of the treatment to the patients. Our results, although they cannot be generalized due to the small sample size, indicate that GH treatment may be beneficial even in those patients who have a positive genetic study, which often limits the growth hormone treatment.

Therefore, this reinforces the fact that the genetic analysis of SGA patients is complex, and that a periodic reassessment of the original diagnosis in these patients is crucial, due to their broad heterogeneity and their need for precision medicine.

On the other hand, an in-depth study of puberty in SGA patients will help us determine which patients may require treatment with GnRH analogs or even aromatase inhibitors, because the loss of height gain during puberty is marked in all SGA patients and probably not only those who present a poor height prognosis may benefit from a combined treatment. Future studies are needed to compare the treatment response in patients receiving aromatase inhibitors and GH treatment versus those receiving GH treatment alone.

## Figures and Tables

**Table 1:** Initial descriptive variables.

Initial Auxological Variables			
Characteristcs	N	Mean	SDS
Weight at birth (SDS)	44	−2.34	0.84
Height at birth (SDS)	44	−2.89	0.66
Target height (SDS)	44	−0.97	0.79
Height at start of treatment (SDS)	44	−3.3	0.64
Age at start of treatment (years)	44	6.4	2.49
Distance to mid-parental height (DMPH) [initial height − target height] (SDS)	44	−2.2	0.92

**Table 2:** Evolutionary variables after 1 and 3 years of treatment.

Evolutionary Auxological Variables at First and Third Year of Treatment						
Characteristics	N	Mean	SDS	Boys (n = 17)	Girls (n = 21)	*p* (Statistical Significance)
Height after 1 y of treatment (SDS)	44	−2.5	0.7	−2.3 (0.5)	−2.6 (0.9)	0.06
Height gain after 1 y of treatment (SDS)	44	0.71	0.6	0.87 (0.8)	0.54 (0.8)	0.6
Weight after 1 y of treatment (SDS)	39	−1.5	0.41	-	-	
BMI after 1 y of treatment (SDS)	39	−0.9	0.56	-	-	
Height after 3 y of treatment (SDS)	38	−1.8	0.89	−1.65 (0.60)	2.02 (1.08)	0.24
Height gain after 3 y of treatment (SDS)	38	1.3	1.2	1.4 (0.55)	1.2 (0.6)	0.49

**Table 3:** Pubertal variables compared by sex.

Pubertal Data of SGA Children Who Reached Adult Height					
Characteristics	N	Mean	Boys (n = 15)	Girls (n = 19)	*p* (Statistical Significance)
Age of puberty onset (years)	29	9.94 (1.01)	10.48 (1.16)	9.94 (0.8)	0.36
Height at puberty onset (SDS)	29	−1.3 (0.97)	−0.99 (0.84)	−1.68 (0.99)	0.33
Total pubertal gain/TPG (cm)	29	19.25 (6.7)	24.14 (5.2)	14.73 (4.3)	0.000

**Table 4:** Adult variables compared by sex.

Adult Variables					
Characteristics	N	Mean	Boys (n = 12)	Girls (n = 17)	*p* (Statistical Significance)
Adult height (SDS)	29	−1.59 (1.12)	−1.2 (1.03)	−1.83 (1.16)	0.25
Total height gain (SDS)	29	1.69 (0.88)	1.81 (0.93)	1.60 (0.87)	0.75
Adult DMPH (SDS)	29	0.65 (1.15)	0.51 (1.25)	0.76 (1.1)	0.9

**Table 5:** Spearman’s linear correlation.

	Adult Height (SDS)		Total Height Gain (SDS)		Total Pubertal Gain (SDS)	
	*R*	*p*	*R*	*p*	*R*	*p*
Height at birth	−0.36	0.05	−0.23	0.15	−0.35	0.1
Weight at birth	0.26	0.9	−0.08	0.6	−0.2	0.24
Target height	0.3	0.15	0.27	0.2	0.33	0.1
Age of onset of GH	−0.17	0.3	−0.6	0.3	−0.22	0.8
Height at start of treatment	0.6	0.02	0.7	0.6	0.1	0.6
DMPH	0.37	0.88	0.22	0.35	−0.1	0.67
Weight at start of treatment	−0.7	0.7	−0.1	0.6	−0.1	0.65
Growth rate at start of treatment	0.03	0.87	0.074	0.67	0.27	0.2
Height gain after 1 y of treatment	0.08	0.5	0.1	0.9	−0.34	0.037
Height gain after 3 y of treatment	0.4	0.048	0.1	0.7	0.24	0.32
Age of onset of puberty	0.2	0.36	0.18	0.38	−0.35	0.51
Height at onset of puberty	0.8	0.01	0.3	0.87	0.29	0.016

**Table 6:** Clinical characteristics of patients with a positive genetic analysis.

	Sex	Height (SDS)	Weight of Newborn (SDS)	Target Height (SDS)	Height at Start of Treatment (SDS)	Age at Start of Treatment (yrs)	Adult Height (SDS)
SGA 1	F	−3.7	−2.65	−2.4	−4.17	9.2	−2.62
SGA 2	M	−2.6	−1.6	−2.4	−3.8	4.3	-
SGA 3	M	−2.7	−3	0.09	−2.63	7.1	−1.5
SGA 4	M	−3.2	−2.79	−2.06	−3.3	9.9	
SGA 5	F	−2.8	−1.02	−2.1	−3.8	7.9	−1.9
SGA 6	F	−2.9	−1.4	−1.76	−3.44	7.6	-
SGA 7	M	−3.1	−2.5	(adopted)	−3.4	3.6	-
SGA 8	F	−2.61	−0.5	0.9	−3.6	5.3	

**Table 7:** Variants found in the patients analyzed. PV: pathogenic variant; VUS: variant of uncertain significance; LPV: likely pathogenic variant.

	Height before GH	Gene	DNA Changes	Protein Changes	Classification	OMIM Ref.
SGA 1	−4.17	*ACAN* NM_001369268.1	c.2764delG	p. Asp922Metfs*23	PV	#608361, #165800, #612813
SGA 2	−3.8	*ACAN* NM_001369268.1	c.2764delG	p. Asp922Metfs*23	PV	#608361, #165800, #612813
SGA 3	−2.63	*TRRAP* NM_001375524.1	c.3884A>G	p. His1295Arg	VUS	#618778, #618454
SGA 4	−3.3	*IHH* NM_002181.4	c.229C>A	p. Arg77Ser	VUS	#607778, #112500
SGA 5	−3.8	*FGFR3* NM_000142.5	c.2318G>T	p. Gly773Val	VUS	#100800, #109800, #610474, #612247, #616482, #187600
SGA 6	−3.44	*GHR* NM_000163.5	c.409G>A	p. Asp137Asn	VUS	#604271, #262500, #143890
SGA 7	−3.4	*GHR* NM_000163.5 *ACAN* NM_001369268.1	c.182G>A c.1948G>A	p. Arg61Gln p. Val650Met	VUS LPV	#604271, #262500, #143890 #608361, #165800, #612813
SGA 8	−3.6	IGF1R NM_000875.5	del15q26.2-q26.3	-	PV	#270450

## Data Availability

The data presented in this study are available upon request from the corresponding author. The data are not publicly available due to privacy.
